# On the 400th anniversary of the birth of Thomas Willis

**DOI:** 10.1093/brain/awab016

**Published:** 2021-01-29

**Authors:** Zoltán Molnár

**Affiliations:** Department of Physiology, Anatomy and Genetics, University of Oxford, Parks Road, Oxford OX1 3PT, UK

## Abstract

Zoltan Molnar marks the 400th anniversary of the birth of one of the great neuroanatomists, Thomas Willis. While Willis’ name is usually associated with ‘the circle’ and the word ‘neurologia’, his work also formed the foundation of modern translational research and clinical medicine.

***In 2021 we celebrate the 400th anniversary of the birth of one of the greatest neuroanatomists, Thomas Willis, the founder of clinical neurology. Willis’ name is usually associated with ‘the circle’ and the word ‘neurologia’, but his work, which comprised insightful descriptions of clinical cases and clear, well-illustrated and articulated scientific publications and case histories, also formed the foundation of modern translational research and clinical medicine. In the 16th and 17th centuries classical medical training was based on academic, scholastic tradition, not empirical observations. Willis’ work highlights the importance of first-hand clinical observations, and the personalized care of patients and their families. It also emphasizes the importance of interdisciplinary working and the significance of different viewpoints.***

Willis’ family originated from a village a few miles north-west of Oxford where they farmed as tenants of St John’s College Oxford. He was born on 27 January 1621 in Ivy Cottage, Farm Lane, a house that is still standing today. A few years later his mother, Rachel, inherited some land just 2 miles away from Oxford at North Hinksey where the family moved to stay in Ferry Cottage. Willis was only 10 years old when she died. From North Hinksey Willis walked to his school, Sylvester Academy, off High Street in the centre of Oxford. This school gave him excellent proficiency in Classics and Latin, and prepared him for his matriculation into the University of Oxford in 1638 at the age of 16.^1^

He received a classical education in the liberal arts (grammar, logic, rhetoric, geometry, arithmetic, astronomy, and music) and was granted a scholarship for being the first servitor to Canon Dr Thomas Iles at Christ Church, Oxford. Willis assisted Mrs Iles in preparing medicines, and this introduction to science most probably influenced his subsequent career choices. At Christ Church he was thoroughly instructed in the classics, grammar, rhetoric, and logic with all instruction in Latin and Greek, receiving his BA on 12 June 1639.^1^

Most physicians at the time had a medical degree from either Oxford or Cambridge Universities. To gain such a qualification one had to put in the years to memorize Aristotle and Galen without much critical thought, although it was also possible to gain a medical degree by royal command or on conferral by the Archbishop of Canterbury. It was customary to then study at Paris, Leiden, Padua, Basel, or Montpellier and on return to be awarded an Oxford degree. Willis began his medical studies after he obtained his MA in 1642. However, in the very same month the English Civil War broke out. The political environment with the Protectorate and the Commonwealth, and subsequent Restoration, shaped Willis’ life. In spite of Oxford being in turmoil, this was also a period of enormous development in many disciplines, including chemistry, physics, astronomy, histology and anatomy (Supplementary Videos 1 and 2).^2^

Ironically, Willis’ medical career benefited from the political upheaval, which saved him from the detrimental effects of regurgitating outdated works and freed him to make accurate and original observations in several fields, especially in the study of the nervous system. Because of the political circumstances in Oxford he was largely spared the stifling effect of a traditional medical education. In 1644, Oxford hosted the King at Christ Church and Queen at Merton College. Oxford became a garrison town, and was under constant siege, with the plague spreading across soldiers and the population. Willis’ medical education was cut short; he joined the royalists, and was later rewarded for his loyalty by being awarded in December 1646, after only 6 months of study, a Bachelor of Medicine degree by Charles I.^1^

From this point Willis was licensed to practice medicine. His medical education had been cut short, giving him the intellectual freedom to gain knowledge from his own observations and clinical experience. He explored a vast number of clinical cases with an open mind and searched for rational explanations for diseases.^3^ After the Restoration of the monarchy by King Charles II, Willis again found favour, and in 1660 received his Doctor of Medicine degree, and was appointed to the Sedleian Chair of Natural Philosophy at Oxford.

Records suggest that as a young doctor Willis started practicing in neighbouring towns and villages and travelled to markets to attract patients in a 50-mile radius around Oxford.^3^ He had to share a horse with one of his colleagues, and not only had to compete with other doctors, but also with quacks and charlatans who had no medical degree. His casebook provides a very good insight into the patients he encountered and the symptoms that led him to his diagnoses.^3^ The descriptions are so accurate that they could be used for teaching even today.

John Locke (1632–1704), who was a philosopher and medical researcher, took notes on Willis’ lectures, and his notes, together with those of Richard Lower, who was Willis’ student, were the basis of Willis’ Oxford Lectures.^3^ His teaching and original clinical research was disseminated beyond Oxford through his publications (Supplementary Videos 3 and 4).^1^^,^^4^^–^^6^ Willis’ impact on clinical medicine spread across Europe. His accounts of diseases are highly insightful; he paid attention to minute details and collections of symptoms that led him to deep insights into medical conditions, such as achalasia of the cardia, myasthenia gravis, paracusis Willisii, asthma and emphysema, diabetes mellitus, dementia, encephalitis lethargica, malaria, typhoid fever, puerperal fever, neurosyphilis, narcolepsy, tuberculosis, and pulmonary diseases (Supplementary Video 5).^1^^,^^3^^,^^5^^–^^7^

Willis’ notes and correspondence with his colleagues reveal a caring and dedicated clinician following his patients’ progress^8^ (Fig. 1 and Supplementary Video 4) (see online exhibition at St John’s College at https://sway.office.com/sDxoxop8O0u4gRD3?ref=Link). This correspondence contains his recommendations for treatment, and recipes, usually in Latin, for medicines. He also recommends treatment by other physicians, appearing collaborative and open to working out the best treatment for the patients. Willis was extremely influential in establishing pharmaceutical treatments. He often used mineral waters containing iron and various syrups. His recipes were in general circulation and were published in a collection that attests to the interest in them beyond his death.

**Figure 1:**
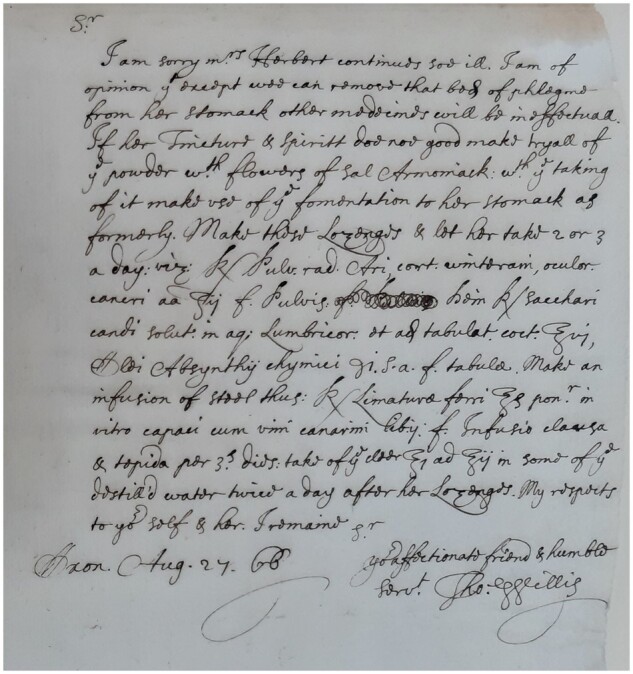
**Letter by Thomas Willis to Richard Higges, 27 August 1666 (St John’s College Library MS 296, fol. 33).** Willis pursues the case of Mrs Herbert, who remains ill. He suggests a body of phlegm to be removed from her stomach and gives advice on medications. Here, too, he includes a Latin recipe (lozenges to be taken two or three times a day). St John’s College library has 15 letters of Thomas Willis, all of them addressed to Dr Richard Higges, a physician based in Coventry. These letters are part of a larger collection of 54 letters written between 1660 and 1690 and mostly addressed to Dr Higges^8^ (Supplementary Video 4 and https://sway.office.com/sDxoxop8O0u4gRD3?ref=Link).

Willis’ account of his patients has clear similarities to the way clinicians think today. His modern view on disease is one of his greatest legacies. The extent of detail about clinical cases, the way he made observations, and his curiosity about the fundamental correlation with anatomy was an extremely new approach at the time (Supplementary Video 5).^4^ He had huge intellectual curiosity; he was not just approaching clinical cases with a set of dogmas. He wished to explore, understand, and find a rational explanation for diseases. His approach to medicine has all the main attributes of a very modern thinker. Willis transformed clinical medicine, from a culture that had not changed for 1000 years and did not question authority into one which was characterized by a questioning attitude and an approach based on empiricism, observation, and on anatomical and clinical correlation, the basis of how we still approach clinical problems today.

The time of the Civil War, Protectorate, and Restoration brought some of the greatest scientific breakthroughs of the period. Historic turmoil transformed and reformed views and ways of working, study and interaction. Science tried to explain things in harmony with religion and these new ways of thinking grew inside the more traditional ones (Supplementary Videos 1 and 2).^2^ Thomas Willis was part of a Royalist group that included high-church clergymen who conducted formal religious services. After 1648, Willis hosted illegal services in Latin in his college rooms at Christ Church and then in Beam (or Beaham) Hall (Supplementary Video 6).

In 1660 Willis started brain dissections, and it is believed that his shift towards neurology was religiously motivated.^9^ He wanted to find the relationship between soul and body. He compared the nervous systems of many different organisms and believed that by understanding differences one could understand what he described as the three forms of soul—vital, rational and immortal. According to Willis, only humans had rational and immortal soul. He had no dilemmas combining his work with his religion (Supplementary Videos 1 and 2). He was a traditional conservative and pious Anglican, but he fully embraced empirical practices, which at the time would have been seen as rather unusual. Willis wanted to understand the soul, but within the framework of trying to understand how the body works and how it relates to anatomy.

It is not a coincidence that these intellectual fermentations occurred during the Civil War. By 1660 Oxford admitted around 400 students (BA 240, MA 150). This large group of young scholars was stranded. Now they had a lot of time on their hands. They could work in teams, freely experiment and satisfy their curiosities (Supplementary Video 2). It is no coincidence that it was at this time a small collaborative team was established. John Wilkins (philosopher and theologian, Warden of Wadham College), Robert Boyle (chemist, inventor of the air pump), John Wallis (mathematician and linguist) and Willis were at the centre of the ‘Oxford Philosophical Club’, 1649–60, from which the Royal Society formed after a lecture given by Christopher Wren in London on 28 November 1660.^1^^,^^5^

Willis’ mentality reflects the founding idea of the Royal Society:

‘I determined with my self seriously to enter presently upon a new course, and to rely on one thing, not to pin my faith on the received Opinions of others, nor on the suspicions and guesses of my own mind, but for the future to believe Nature’

The motto of the Royal Society is ‘*Nullius in verba’*, ‘take nobody’s word for it’.^9^

The dissections were conducted in Christ Church, Willis’ home and laboratory in Beam/Beaham Hall, on Merton Street (see Stuart Panter’s video on Thomas Willis’ Works Rooms at https://www.youtube.com/watch?v=hsrJd3V3A) and in Petty’s lodgings at Bulkley Hall, off High Street at the site where the Thai restaurant, Chiang Mai Kitchen, is situated today. The first observations were published in *Cerebri Anatome* (1664). Willis’ anatomical observations were based largely on his personal, clinical, and autopsy experience from the dissection of his own patients when they died.^7^ This allowed him to correlate his clinical observations with pathological changes. For example, he noticed that a young male who exhibited signs of intractable epilepsy and learning difficulties had more, but smaller sulci and gyri on his cerebral cortex. He associated these alterations of the cortical morphology with the neurological conditions and developed original hypotheses about the mechanisms of nervous system function.^10^ Willis discussed his observations, attempting to correlate them with the symptoms, signs and mechanisms of disease. All this was completely unheard of before his time (Supplementary Video 5).

Willis also worked with exceptional colleagues. He performed dissections with Sir William Petty (physician, mathematician, and engineer), Thomas Sydenham (physician), Thomas Millington (physician), John Locke (physician and philosopher), Edmond King (anatomist, physiologist, and clinician) (Fig. 2 and Supplementary Videos 2 and 6). The output of this team is staggering, and they laid the foundations of numerous disciplines. Robert Hooke, one of Willis’ assistants in the late 1650s, later coined the term ‘cell’ in his *Micrographia* in 1665 (https://sway.office.com/sDxoxop8O0u4gRD3?ref=Link). The famous architect Christopher Wren collaborated with Willis on the illustrations of *Cerebri Anatome* and *De Anima Brutorum*, and made some fundamental contributions to architecture (St Paul’s Cathedral, Sheldonian Theatre, All Souls College).

**Figure 2:**
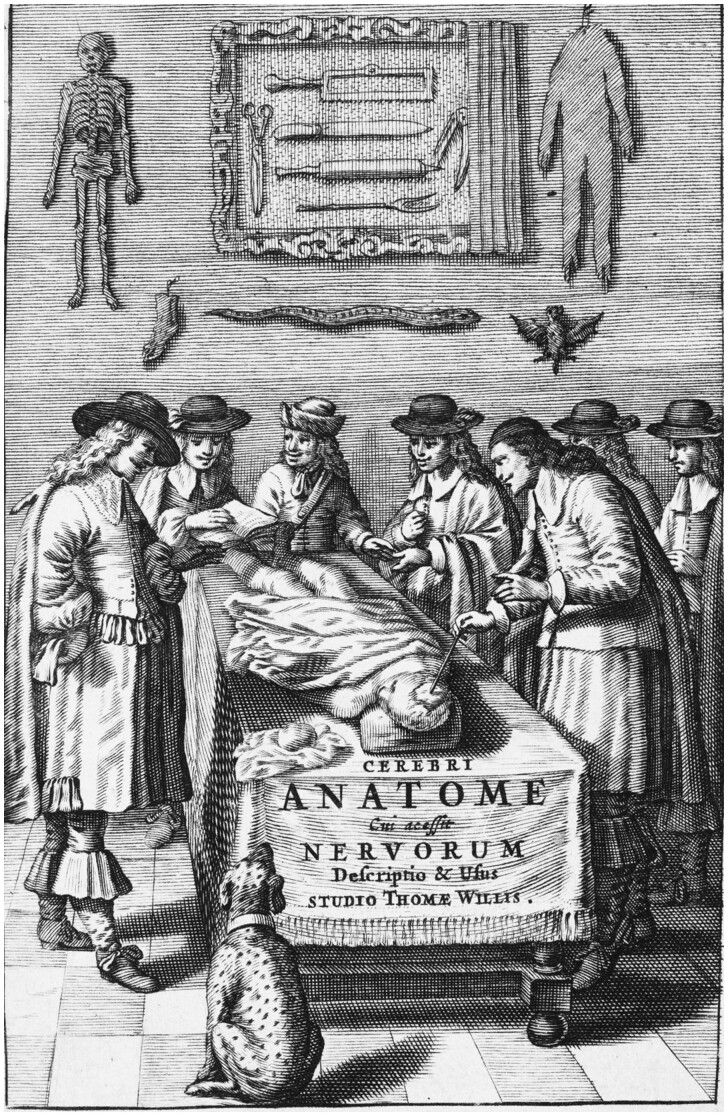
**Title page of *Cerebri Anatome* (2nd ed, 1664) illustrates Willis working as part of a team.** This group comprised Richard Lower, Thomas Millington, Edmond King and Christopher Wren. These associates were involved in dissections, removal and fixation of brains for study, description, and illustration. Willis acknowledged the help of Lower and Wren in his preface in *Cerebri Anatome*. Courtesy of Cushing/Whitney Medical Library, Yale University.

Another protégé of Thomas Willis was the medical doctor Richard Lower, who subsequently published on circulation in the heart and performed the first blood transfusion. We know from letters from Robert Boyle (chemist) that he was involved in the generation of ‘fixative’ from port wine and vinegar to prolong the life of the brain tissue after dissection. The intellectual energy emanating from all of these scholars was extraordinary (Supplementary Video 2). After the Restoration most were awarded titles to reward their loyalty to the Crown.

Thomas Willis made pioneering observations of various neural structures, and his system of nomenclature is still in use today (Supplementary Video 7). He is credited with naming several structures in the brain: corpus striatum, internal capsule, cerebellar peduncles, anterior commissure, claustrum, inferior olives, pyramids, optic thalamus, spinal accessory nerve, stria terminalis, vagus nerve, intercostal nerve (sympathetic ganglionic chain), and ophthalmic nerve.^1^ Willis placed the thalamus underneath the striated bodies at the top of the medulla oblongata. He noted that there was no cavitation in the thalamus as Galen originally described. Although it is clear from Willis’ illustrations of the cut cranial nerves that he identified all of them, his numbering distinguished only nine pairs, because he combined nerves VII and VIII together as ‘VII’, and IX, X, and XI together as ‘VIII’.^9^^,^^11^

Willis is credited with the discovery of the arteriosus circle at the base of the brain, which bears his name today, the circle of Willis (Supplementary Video 8), although he was not the first to describe this structure. He also described a compelling case report of a man who died of a stomach tumour. When he dissected the patient he noticed that the right intracranial artery was sclerotic and completely occluded. He noted that in spite of this blockage of blood flow the patient had no neurological deficits (Supplementary Videos 5 and 8). He also noticed the compensatory dilation of the ipsilateral vertebral artery and used mercury dyes to detect the anastomotic vessels.^1^^,^^3^

Willis also conceptually dissociated voluntary movements that originate in the cortex, from involuntary ones that originate in the cerebellum (Supplementary Video 3).^5^ He made the distinction between voluntary and what he called reflexive movement. This is the concept of the reflex and he was very much interested in those parts of the nervous system that are primarily concerned with what we now call vegetative function, in other words he provided the first articulation of the autonomic nervous system (Supplementary Video 3).^4^^,^^5^ Willis was also the first person to propose that the higher cognitive function of the human brain comes from the convolutions of the cerebral cortex. He based his argument on his comparative work and his observations on cortical malformations associated with learning difficulties.^8^^,^^10^^,^^12^

Willis and Petty probably also discovered neuroprotection. A young female, Anne Green, who was probably hypoglycaemic and certainly hypothermic, was resuscitated after a failed hanging.^13^ The resuscitation was successful despite the prolonged duration and the fact that she most likely had some kind of cessation of cerebral perfusion.^8^ The reason she recovered was what we now understand to be key to physiological protection of the brain: a drop in glucose and a drop in temperature. This was discovered using animal models of resuscitation some 400 years later. Willis was probably the first person to perform an effective resuscitation unwittingly (Supplementary Video 8).

Willis’ way of observing and treating patients was different to that of most of his contemporaries, owing to his original observations and critical views. His experiments marked the transition between medieval and modern notions of brain function, and possessed the essence of translational research, which attempts to connect medical innovation directly to patient care (Supplementary Video 5). Dissecting his own clinical cases was undoubtedly a key element that contributed to his success. He was in a position to relate altered behaviour to abnormalities of the brain. He recognized the pathological basis of several diseases, and his anatomical descriptions and case histories remain valid today. By combining insightful clinical observations with original pathological studies, his enquiring mind established links that are still astonishing 400 years on. For these reasons, Willis and his extraordinary achievements deserve to be described to every new generation of anatomists and neuroscientists.

## Notes

*Zoltán Molnár is Professor of Developmental Neuroscience at the Department of Physiology, Anatomy and Genetics and Tutorial Fellow in Human Anatomy at St John’s College, University of Oxford (https://www.dpag.ox.ac.uk/team/zoltan-molnar). He initiated the History of Medical Sciences Project website at the University of Oxford (https://history.medsci.ox.ac.uk).*
